# Immune dysregulation and exhaustion in EBV- associated hemophagocytic lymphohistiocytosis: insights from single-cell transcriptomics and TCR repertoire analysis

**DOI:** 10.3389/fimmu.2026.1868027

**Published:** 2026-08-05

**Authors:** Xiaodan Luo, Ao Chen, Jianbo Liu, Boyun Shi, Jianli Tang, Jiayu Huang, Danyun Yuan, Zhiming Liang, Huo Tan, Runhui Zheng

**Affiliations:** The Fifth Affiliated Hospital, Guangzhou Medical University, Guangzhou, China

**Keywords:** EBV, hemophagocytic lymphohistiocytosis, immune dysregulation, single-cell RNA sequencing, T cell exhaustion

## Abstract

**Introduction:**

Epstein-Barr virus-associated hemophagocytic lymphohistiocytosis (EBV-HLH) is a life-threatening hyperinflammatory syndrome paradoxically characterized by excessive cytokine production and immune activation despite failure to clear Epstein-Barr virus (EBV). The cellular mechanisms underlying immune dysregulation in EBV-HLH remain incompletely understood.

**Methods:**

We performed single-cell RNA sequencing (scRNA-seq) and single-cell T-cell receptor sequencing (scTCR-seq) to characterize the peripheral immune landscape of patients with EBV-HLH. Key findings were validated by flow cytometry and enzyme-linked immunosorbent assay (ELISA) in an independent cohort of 37 patients with secondary hemophagocytic lymphohistiocytosis (sHLH).

**Results:**

We identified EBV transcript-positive NK/NKT-like cells undergoing clonal expansion and proliferation as key contributors to immune dysfunction. These cells exhibited high CD160 expression, increased interferon-γ expression, and enrichment of IL-10-mediated signaling. In addition, monocytes acquired an immunosuppressive phenotype characterized by enhanced TGFB1 expression and increased migratory and phagocytic functions. This dysregulated immune environment may promote CD8^+^ T-cell exhaustion and abnormal clonal expansion of effector CD4^+^ T cells, ultimately contributing to EBV tolerance.

**Discussion:**

This study delineates the peripheral immune landscape of EBV-HLH and identifies potential therapeutic targets for EBV-HLH.

## Graphical Abstract

**Figure:**
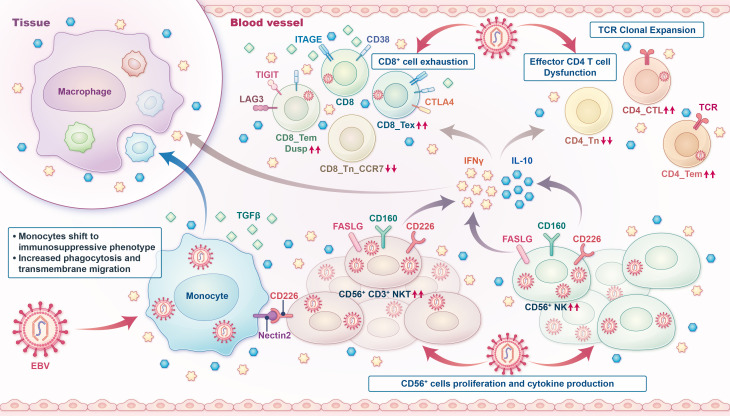
The Peripheral Immune Landscape of EBV-HLH. EBV transcript-positive cytotoxic lymphocytes, including NK cells and NKT-like/CD56^+^ cytotoxic T-lineage cells, show CD160 expression, clonal expansion, and activation, accompanied by increased IFNG expression and elevated systemic IL-10 levels. Monocytes display an immunoregulatory phenotype with increased TGFB1 expression, migration-related programs, and phagocytic activity. Together, these immune alterations are associated with CD8^+^ T-cell exhaustion and effector CD4^+^ T-cell expansion, potentially contributing to impaired EBV clearance.The abstract should ideally be structured according to the IMRaD format (Introduction, Methods, Results and Discussion). Provide a structured abstract if possible. If your article has been copyedited by us, please provide the updated abstract based on this version.

## Introduction

Hemophagocytic lymphohistiocytosis (HLH) is a relatively rare and life-threatening hyperinflammatory syndrome that can occur in patients with genetic defects or arise in individuals with malignancies or underlying infections, often associated with Epstein-Barr virus (EBV) infection (1, 2). EBV-associated HLH (EBV-HLH) can either be non-neoplastic in origin, notably chronic active EBV disease (CAEBV), or neoplastic, associated with EBV^+^ T/natural killer (NK) cell lymphomas. Impaired NK cell function, hyperactivation of macrophages and T cells are prominent characteristics of HLH (2, 3). However, this hyperactivation leads to severe systemic inflammation and organ damage rather than effectively eliminating causes such as EBV. The treatment response of EBV-HLH is usually transient with chemotherapy alone, and disease relapse or progression is often associated with EBV reactivation, which complicates treatment strategies. Allogeneic hematopoietic stem cell transplantation (allo-HSCT) is considered the only curative treatment for EBV-HLH; however, a subset of patients may still experience disease relapse after allo-HSCT, often due to persistent EBV reactivation (2, 4).

EBV infects over 90% of the global population and persists for the lifetime of the person (5). Various clinical presentations, from asymptomatic infection to lethal HLH, suggest that the host’s immune microenvironment plays a pivotal role in the outcome of EBV infection (3, 6). The immune cell landscape of EBV-HLH remains poorly understood. T cell exhaustion is a well-documented phenomenon in chronic viral infections and various forms of cancer (3, 7, 8). Exhausted CD8^+^ T cells often exhibit dysfunctional states, with alterations in T-cell receptor (TCR) signaling, differential expression of transcription factors (TFs), elevated levels of inhibitory immune checkpoints and reduced effector cytokine production (3, 7, 9). However, in the context of EBV-HLH, the specific role of immune cell exhaustion remains unclear. This study explores the immune cell landscape and progression trajectory in patients with persistent EBV infection through single-cell RNA sequencing (scRNA-seq) paired with single-cell TCR sequencing (scTCR-seq), focusing on immune functions and interactions of NK cells, CD8^+^ T cells, and myeloid cells across disease stages. We aimed to identify potential mechanisms underlying the failure to clear EBV and EBV-HLH pathogenesis, which may have implications for identifying high-risk populations and improving therapeutic strategies.

## Methods

### Patient enrollment and blood sample processing

**Figure 1:**
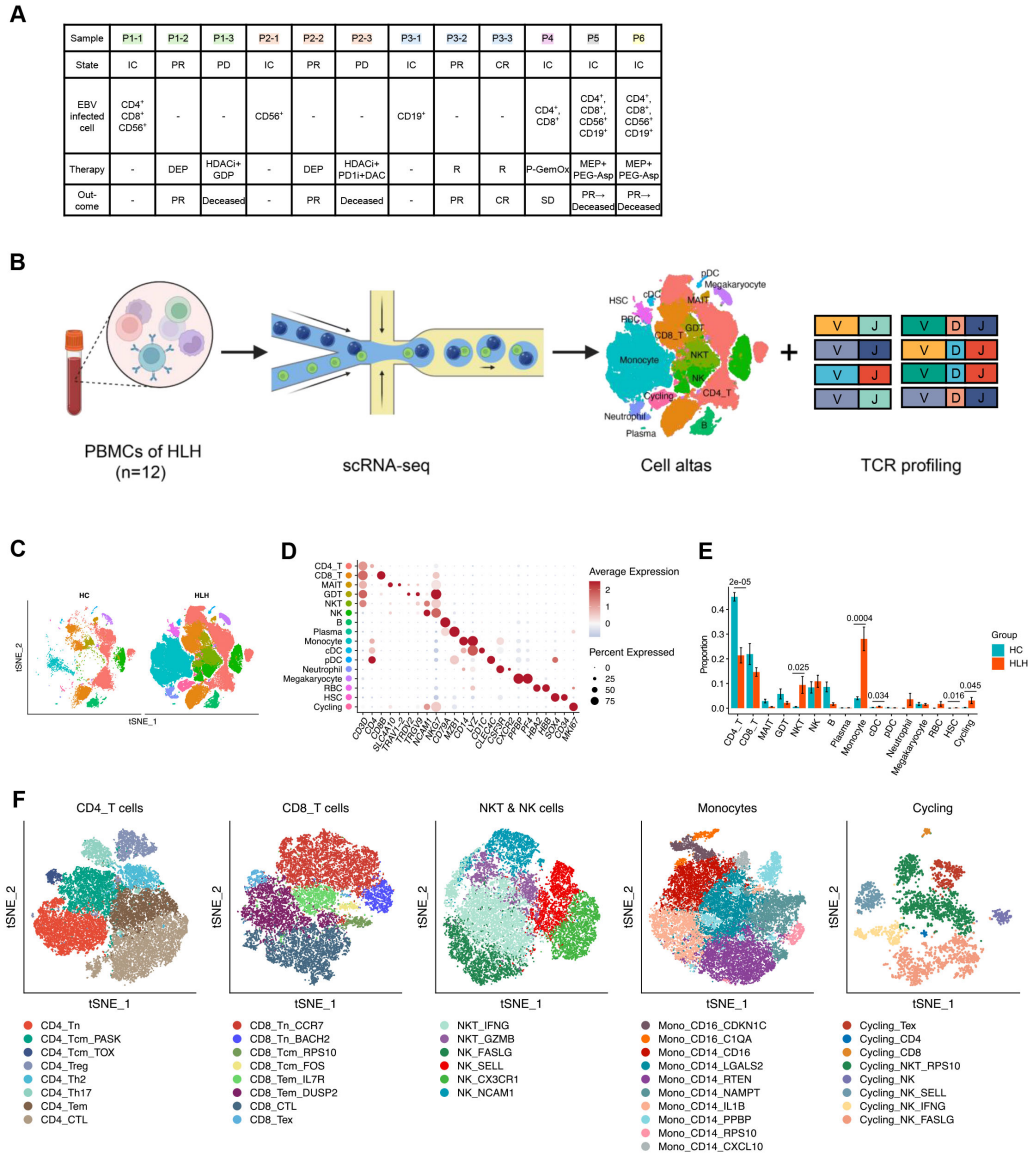
PBMCs classification and characterization in EBV-HLH patients by scRNA-seq. **(A)** Clinical information of the recruited EBV-HLH patients for single-cell sequencing analysis (scRNA-seq). For State or Outcome, IC, Initial condition; PR, partial response; PD, progressive disease; CR, complete response; SD, stable disease. For Therapy, DEP: Liposomal doxorubicin, etoposide, and high-dose methylprednisolone; HDACi, Histone Deacetylase Inhibitor; GDP, Gemcitabine, Dexamethasone, Cisplatin; PD1i, Programmed Death-1 inhibitor; DAC, Decitabine; R, Rituximab; P-GemOx, PEG-aspargase, Gemcitabine, Oxaliplatin; MEP: Mitoxantrone liposomes, etoposide and high-dose methylprednisolone; PEG-Asp: PEG-aspargase. **(B)** Study design schematics. PBMC samples from six EBV-HLH patients were analyzed by scRNA-seq for transcriptome profiling, and single-cell T-cell receptor sequencing (scTCR-seq) data were available for three of these patients for TCR repertoire analysis. Public scRNA-seq and scTCR-seq data from 3 HCs were used for comparative analyses. **(C)** tSNE (t-distributed Stochastic Neighbor Embedding) plot of the PBMC scRNA-seq data, split by sample group. HC: healthy control; HLH: EBV-HLH patient. **(D)** Dot plot for selected marker genes (x-axis) of the identified major cell types (y-axis). Color scale indicates the mean of normalized expression of genes in each cell type, and dot size is proportional to the percentage of cells within each cell cluster expressing the genes. The same applies to all dot plots in this paper, unless stated otherwise. **(E)** Bar chart for comparing the abundance of each identified major cell type between HC and HLH donors. Data are shown as mean±sem. *P*-values (t-test) for cell types with significant inter-groups difference are shown on top of the corresponding bars. **(F)** tSNE plots for each of the major cell types in PBMC. Cells in each cell type were re-clustered according to scRNA-seq data for subtype delineation. Cell subtype suffix indicates the corresponding marker gene.

Six EBV-HLH patients were enrolled in this study. P1–3 were from previously studies (10), while P4–6 were newly recruited. Samples from P1–3 were collected at diagnosis, remission and relapse/progression. All enrolled patients fulfilled the HLH-2004 diagnostic criteria for EBV-HLH (11). Their underlying diagnoses and clinical characteristics are summarized in **Figure 1A**; **Supplementary Table 1**. Public scRNA-seq and scTCR-seq data from 3 age-matched healthy controls (HCs) (F012, F013 and F014) was obtained from GEO (GSE157007) (12).

Fresh peripheral blood was collected in EDTA-treated tubes. Peripheral blood mononuclear cells (PBMCs) were isolated by Ficoll Pague (GE Healthcare Life Sciences) density gradient centrifugation. Red blood cell lysis was performed using standard lysis procedures. Cell concentration and viability were assessed prior to downstream analyses.

### Single-cell RNA-sequencing experiment

Cell number and viability were assessed following surface protein staining, with a viability threshold of >80%. Droplet-encapsulation single-cell sequencing was then performed, with 10,000-15,000 viable single cells loaded onto each channel of the Chromium Controller (10x Genomics, RRID: SCR_016654). After droplet encapsulation, single-cell cDNA synthesis, amplification, and library preparation were conducted using the Chromium Single Cell 5′ Feature Barcode Library Kit, Chromium Single Cell 5′ Library & Gel Bead Kit, and Chromium Single Cell V(D)J Enrichment Kit for Human T Cells (all from 10x Genomics). Libraries from each channel were pooled together and sequenced on an Illumina NovaSeq 6000 (RRID: SCR_016387).

### scRNA-seq data processing and cell type annotation

Single-cell gene expression and immune profiling libraries were generated using the 10x Genomics Chromium platform. Raw FASTQ files (read 1 and read 2) for scRNA-seq and scTCR-seq were processed with Cell Ranger software (v.7.1.0, RRID: SCR_017344) suite with the human reference genome GRCh38 and default parameters, including quality filtering, alignment, V(D)J assembly, and TCRαβ pairing sequentially. Expression matrices were generated for subsequent analysis. For the quantification of EBV-associated gene expression at the single-cell level, we used the EBV reference genome NC_007605.1 from UCSC (RRID: SCR_005780). The quantification was carried out using the Cell Ranger software as above, with the UMI >0 threshold used for exploratory EBV transcript mapping. For sample P1-2, quantification of EBV-related genes was not performed due to missing FASTQ files.

To filter out low-quality cells, those with fewer than 200 or more than 10,000 detected genes, or with a mitochondrial UMI rate exceeding 10%, were excluded. The raw count data were normalized, and highly variable genes were selected for each sample using the R package Seurat (13) (v.4.3.0, RRID: SCR_016341). Batch effects were mitigated by integrating all samples through harmonizing ‘anchors.’ Seurat anchor-based integration was used to reduce major batch-driven variation and to facilitate joint cell-type annotation across samples. Disease-associated differences were further interpreted using sample-level patterns where applicable. During data scaling, the number of reads, number of features, and percentage of mitochondrial reads were regressed out. Principal component analysis (PCA) was performed on the scaled data, focusing on the top 2,000 highly variable genes. The first 30 principal components were used for dimensionality reduction and cell clustering through t-SNE (t-distributed stochastic neighbor embedding) or UMAP (uniform manifold approximation and projection). Clustering was achieved using the Louvain Modularity algorithm. Differentially expressed gene (DEG) analysis was conducted to identify genes meeting the following criteria between cell subtypes: Log2 fold change (Log2FC) > 0.25, p-value < 0.05, and minimum percentage of expression (min.pct) > 10%. These DEGs, together with known marker genes, were used to annotate and classify cell populations. Additionally, cells expressing signature genes from two or more immune cell types were identified as doublets and excluded.

### Calculation of functional gene module score

Functional gene modules were calculated at the single-cell level using the *AddModuleScore* function in Seurat. The average expression levels were adjusted by subtracting the aggregated expression of control gene sets. The functional gene modules included markers for various cellular functions: cytotoxicity (*PRF1, GZMA, GZMB, GZMK, GZMH, GZMM*), exhaustion (*PDCD1*, *HAVCR2, LAG3, TIGIT, CTLA4, CD160, BTLA, LAYN, TOX)*, pro-inflammatory (*IFNG, TNF, IL1B, IL6, IL12B, IL12A, IL18, CCL2, HLA-DQA2, HLA-DQA1, HLA-DPB1, HLA-DQB1, CD1C*), and anti-inflammatory (*IL4, IL10, IL13, TGFB1, FCN1, CD163, CD36, S100A9, S100A8, VCAN, CD14, F13A1, APOE, C1QB, C1QA, C1QC*).

### GO and KEGG pathway analysis

Gene Ontology (GO) Biological Process (BP) terms and Kyoto Encyclopedia of Genes and Genomes (KEGG) pathways enrichment analysis for genes was conducted using R package clusterProfiler (14) (v.4.8.1, RRID: SCR_016884). Significant categories were identified using Fisher’s exact test, with *p*-values adjusted for multiple testing using the Benjamini-Hochberg correction. KEGG pathway activity estimates were also obtained using the GSVA (15) (v.1.42.0, RRID: SCR_021058), which calculates enrichment scores for the gene expression matrix. The *lmFit* function from the R package limma (v.3.50.3, RRID: SCR_010943) was then used to identify significantly different pathways (16).

### Single-cell trajectory inference

For CD4^+^ and CD8^+^ T cell subtypes, partition-based graph abstraction (PAGA) analysis in the Python package Scanpy (v.1.9.3, RRID: SCR_018139) was used to reconstruct cell development trajectories (17, 18). PAGA characterized the connections between cell subpopulations and inferred transitions among different T cell subsets. Naïve T cells were designated as the root cells for the trajectory analysis.

### Single-cell regulatory network inference and clustering analysis

Regulatory network inference was performed using SCENIC (RRID: SCR_017247). The analysis involved four steps (19): (1) single-cell gene expression data for each macrophage subtype was analyzed with a list of 1,390 known human TFs, identifying gene sets co-expressed with TFs via random forest models; (2) putative target genes in each module underwent cis-regulatory motif analysis, retaining modules with significant motif enrichment within ±500 bp or ±10 kb of the transcription start site; (3) the AUCell algorithm (RRID: SCR_017923) scored the activity of each regulon per cell, with regulons having an average AUC score ≥ 0.05 kept for further analysis; and (4) regulon specificity scores were calculated for CD8^+^ T cell subtypes.

### Inference and analysis of cell-cell communication

Intercellular communication was evaluated using CellChat (20) (v.1.6.1, RRID: SCR_021946) and NicheNet (21) (v.2.0.4, RRID: SCR_018994). CellChat was applied with the default workflow to model ligand–receptor–mediated communication between cell types. NicheNet was applied to prioritize ligand-receptor pairs potentially regulating gene set of interest including upregulated genes in HLH compared to HCs for monocytes, with the analysis restricted to receptors expressed in at least 10% of monocytes.

### Analysis of scTCR-seq data

Only TCR sequences associated with cells annotated as αβ T cells by scRNA-seq were retained for analysis. The Python package Scirpy (22) (v.0.18.0, RRID: SCR_021249) was used to process the scTCR-seq data. Cells were categorized based on their number of α and β chains using Scirpy’s *tl.chain_pairing* function, classifying them as “No TCR,” “Orphan beta,” “Orphan alpha,” “Single pair,” “Extra alpha,” “Extra beta,” “Two full chains,” and “Multichain.” Only cells with “Single pair,” “Extra alpha,” or “Extra beta” chains were included for further analysis. Clonotypes were defined using Scirpy’s *pp.ir_dist*, t*l.define_clonotypes*, and tl.clonotype_network functions, based on amino acid sequence similarity. The clonotypes were grouped into four categories: “>5,” “3-5,” “2,” and “1,” according to the number of T cells expressing identical TCRs. Scirpy’s pl.alpha_diversity function was used to calculate the diversity of TCR. TCR clonotype similarity across clusters was assessed using Scirpy’s *tl.repertoire_overlap* function, with Jaccard distance-based hierarchical clustering. Finally, potential TCR specificities were annotated by comparing the sequences to known TCR specificities in the vdjDB (RRID: SCR_007002).

### Flow cytometry analysis and cell sorting

Flow cytometry and cell sorting were performed by the Department of Clinical Laboratory, Fifth Affiliated Hospital of Guangzhou Medical University. For the analysis of EBV transcript-positive lymphocyte subsets, PBMCs were collected and T lymphocytes (CD3^+^CD4^+^ and CD3^+^CD8^+^), B lymphocytes (CD19^+^), and natural killer (NK) cells (CD3^−^CD16^+^CD56^+^) were identified using a multicolor flow cytometry assay. Cells were stained with fluorochrome-conjugated monoclonal antibodies (BD Biosciences, San Jose, CA, USA) and analyzed using a BD FACSMelody flow cytometer (RRID: SCR_018890) following the manufacturer’s protocol. Sorted subsets were analyzed for EBV-DNA copy number by qPCR. Data acquisition and analysis were performed using BD FACSChorus (Tree Star, Ashland, OR, USA, RRID: SCR_018890). Cytokine levels (IL-10, IFN-γ, and IL-1β) were quantified using bead-based immunoassay (CytoSYS, BeamCyte, Changzhou Beidaoke Biotechnology Co., Ltd.). For validation, PBMCs from patients at diagnosis were sorted into CD14^+^ monocytes and CD3^-^/CD3^+^CD56^+^ NK/NKT cells by fluorescence-activated cell sorting. Post-sort purity was assessed and the sorted cells were used for cytokine quantification by enzyme-linked immunosorbent assay (ELISA).

### ELISA

Samples from 5 HLH patients and 5 HCs were analyzed. IL-10 and IFN-γ levels in supernatants from sorted CD56^+^ NK/NKT cells, and TGF-β and IL-1β in supernatants from sorted CD14^+^ monocytes were quantified according to the manufacturer’s instructions.

### Statistical analyses

All statistical analyses were performed using R (version 4.1.0, RRID: SCR_001905) and Python (version 3.7.3, RRID: SCR_008394). Data normality was assessed using the Shapiro-Wilk test. For comparisons between two independent groups, an unpaired Student’s t-test was used when data followed a normal distribution and variances were equal; otherwise, the non-parametric Wilcoxon rank-sum (Mann-Whitney U) test was applied. *p* < 0.05 was considered statistically significant unless stated otherwise.

## Results

### Peripheral immune cell landscape of EBV-HLH

To characterize peripheral immune dysregulation in EBV-HLH, we performed paired scRNA-seq with scTCR-seq and compared them with HCs (**Figures 1A, B**). After quality control, 120,310 cells were retained for analyses (**Supplementary Figures 1A, B**). We identified 16 major immune cell populations (**Figures 1C, D**) including CD4^+^ T (CD4_T), CD8^+^ T (CD8_T), mucosal-associated invariant T (MAIT), γδT (GDT), natural killer T (NKT), natural killer (NK), B, plasma, monocyte (mono), classical dendritic cell (cDC), plasmacytoid DC (pDC), neutrophil, megakaryocyte, red blood cells (RBCs) and hematopoietic stem cells (HSCs). Cycling cells marked by *MKI67* were significantly enriched in EBV-HLH compared with HCs (**Figures 1C–E**; **Supplementary Figures 1C, D**). EBV-HLH patients also had more monocytes and NKT cells, whereas CD4^+^ T cells were reduced. Further sub-clustering revealed distinct functional subtypes within CD4^+^ T, CD8^+^ T, NKT-like and NK, monocyte and cycling cells (**Figure 1F**; **Supplementary Figures 1E–G**).

### EBV transcript-positive CD56^+^ cells exhibit expansion and cytokine production

**Figure 2:**
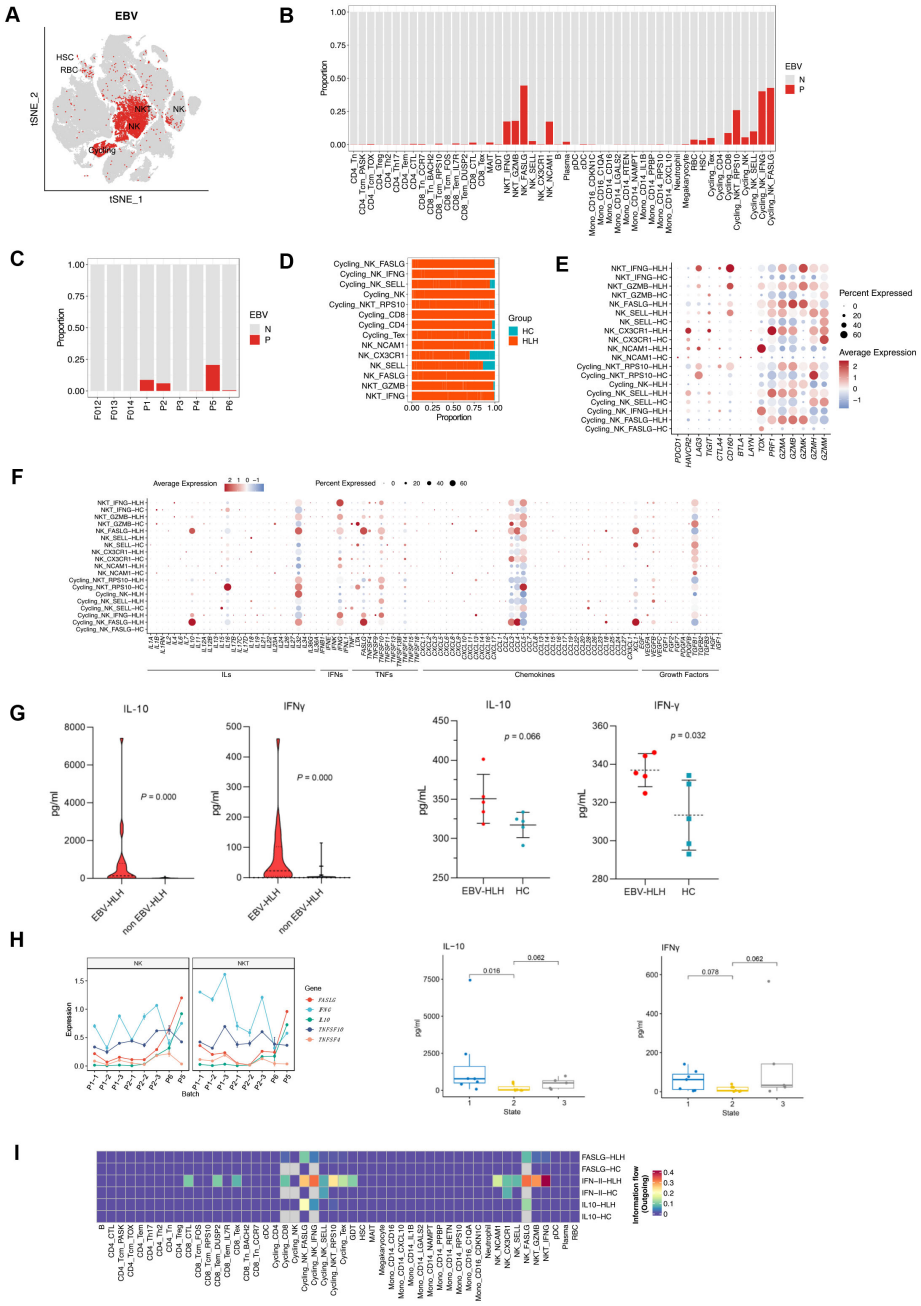
Characterization of NK and NKT cells in PBMC of EBV-HLH patients. **(A)** tSNE plot of all analyzed PBMCs, color-coded according to whether EBV gene expression was detected in the corresponding cell. Red: EBV-positive; gray: EBV-negative. **(B)** Bar chart for summarizing percentage of EBV-positive cells per cell subtype. **(C)** Bar chart for summarizing percentage of EBV-positive cells per donor. **(D)** Bar chart for summarizing percentage of NK, NKT and cycling cells subtype per sample group. **(E)** Dot plot for scaled expression of selected cell exhaustion and cytotoxicity related genes (x-axis) in NK and NKT cell subtypes (y-axis). The last suffix in cell subtype name (HC or HLH) indicates the donor group from which the cells were analyzed. **(F)** Same as E, but for cytokine genes. **(G)** Violin plots comparing plasma IL-10 and IFN-γ levels by multiplex flow cytometry assays in EBV-HLH and non-EBV-HLH patients (left) (Wilcoxon Rank Sum test, two-sided). Dot plots showing IL-10 and IFN-γ secretion in culture supernatants from sorted CD56^+^ NK/NKT cells from EBV-HLH patients and HCs, as measured by ELISA (right). (Independent-samples Student’s t-test). **(H)** Line graphs showing the average expression (y-axis) of selected cytokines in NK and NKT cells of distinct patient samples (x-axis). Sample suffix (1, 2 or 3) corresponds to the disease state (Initial condition, remission or progression). Box plots showing dynamic changes in IL-10 and IFN-γ levels across disease states in EBV-HLH patients. Sample suffix (1, 2 or 3) corresponds to the disease state (Initial condition, remission or progression). **(I)** Heatmap showing the outgoing interaction strength (information flow) from different cell subtypes (x-axis) in HC and HLH donors through selected cytokines (FASLG, IFN-II and IL10). Grey color indicates the cell subtype is not identified in the corresponding donor group.

By mapping the sequenced reads to EBV genome and combining the EBV information with cell subtype, we found that most of the EBV^+^ cells were NK/NKT-like cells (**Figures 2A, B**; **Supplementary Figures 2A, B**), consistent with clinical identification of EBV transcript-positive CD56^+^ cells in patients P1, P2, P5, and P6 (**Figure 1B**; **Supplementary Figure 2A**). Most of the EBV^+^ cells were identified in P1, P2 and P5, especially at the initial and relapse stage, while the HCs (F012, F013 and F014) were not detected with EBV^+^ cells (**Figure 2C**; **Supplementary Figures 2A, B**). Interestingly, P3, P4 and P6 had fewer EBV^+^ cells than the other 3 patients (**Figure 2C**), suggesting limited proliferative expansion in these cases. Consistent with previous reports (23), HSCs exhibited EBV infection and potentially underwent CD34-SELP-mediated chemotactic migration to the peripheral circulation in HLH (**Figure 1E**; **Supplementary Figure 2C**).

Proliferative NK/NKT-like subsets and cytotoxic NKT-like populations (NKT_GZMB and NKT_IFNG) were almost exclusively detected in EBV-HLH, whereas canonical NK subsets (NK_CX3CR1 and NK_SELL) showed minimal EBV infection and were present in HCs (**Figure 2D**). NK/NKT-like cells from HLH patients exhibited higher cytotoxicity and increased exhaustion, particularly marked by the elevated expression of *CD160 (*24, 25) (**Figure 2E**; **Supplementary Figure 2D**). Cytokine profiling revealed increased expression of *IL10*, *IFNG*, *FASLG*, *TNFSF4* and *TNFSF10* in specific NK/NKT-like subsets (**Figure 2F**). To independently validate these key cytokine alterations at the protein level, we analyzed an independent cohort of 37 secondary HLH (sHLH) patients from our center using multiplex flow cytometry and ELISA. Multiplex flow cytometry revealed that IL-10 and IFN-γ levels were significantly elevated in EBV-HLH compared to non-EBV-HLH (Wilcoxon test *P* = 0.000, 0.000, effect size r = 0.59, 0.60, **Figure 2G**), while IL-1β levels did not differ between groups. To further examine the cellular sources of these cytokines, we performed cell sorting followed by ELISA. Sorted CD56^+^ NK/NKT cells from EBV-HLH patients secreted higher levels IFN-γ, whereas IL-10 secretion did not differ between groups despite a higher median level in EBV-HLH (**Figure 2G**). Notably, these NK/NKT-like cells were more abundant during disease progression (**Supplementary Figures 2E, F**), with *IL10*, *IFNG* and *FASLG* expression decreasing during remission and increasing at relapse (**Figure 2H**). As these cytokine gene expression dynamics were derived from the transcriptional profiles of NK/NKT-like cells across all six patients, they were independent of single-cell EBV assignment. Moreover, the NK/NKT-like cells were predicted to have strong outgoing cell-cell interaction signaling in HLH patients (**Figure 2I**). In patient P3 with EBV infection restricted to B cells showed rapid reduction of B cells following rituximab treatment (**Supplementary Figure 1B**, **S2G**).

### Inhibitory function of myeloid cells in EBV-HLH

**Figure 3:**
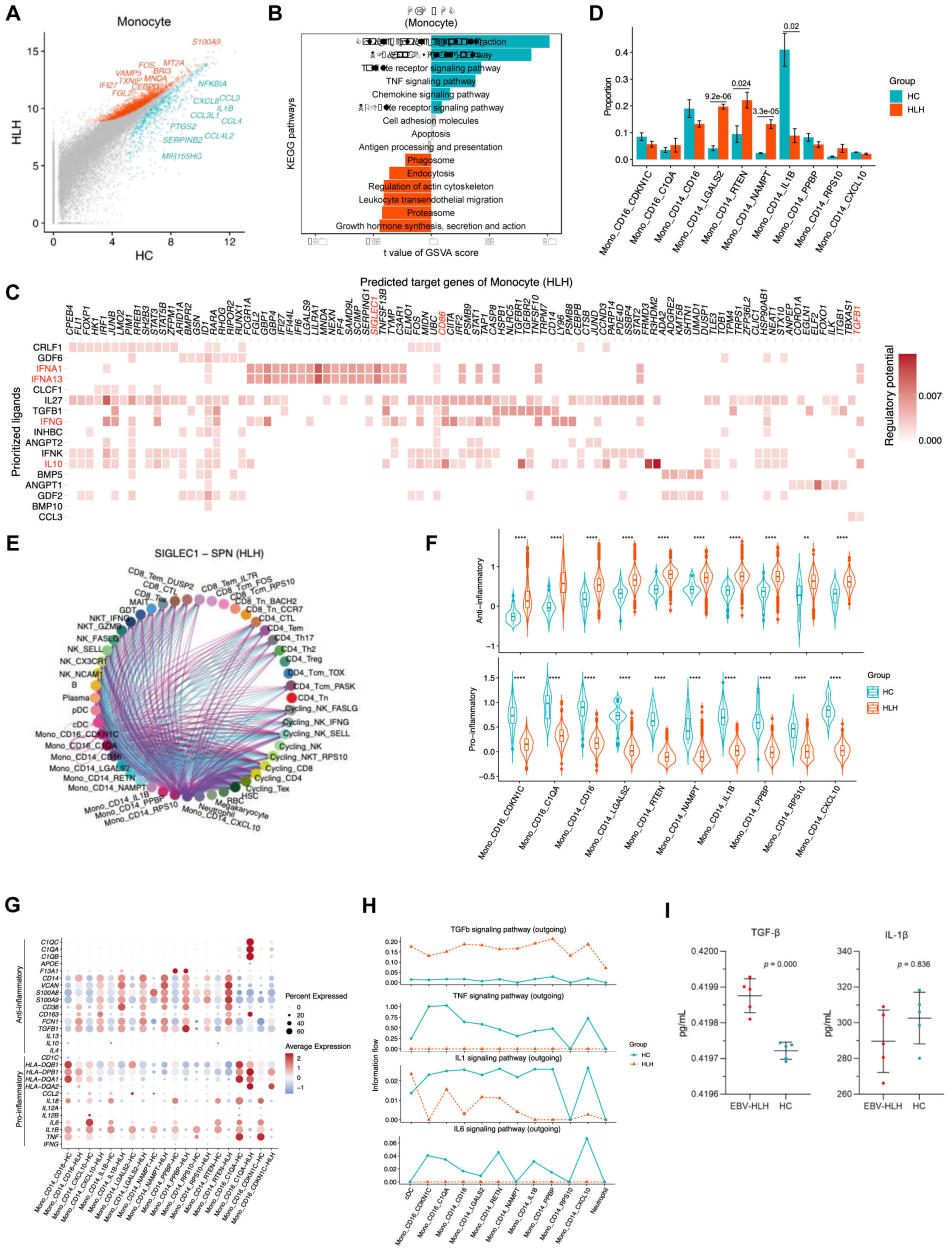
Characterization of myeloid cells in PBMC of EBV-HLH patients. **(A)** Scatterplot for comparing average gene expression in the monocytes of HC and HLH donors. Top 10 genes with significantly upregulated expression in HC and HLH are shown in cyan and orange, respectively. **(B)** Bar chart for the significantly activated Kyoto Encyclopedia of Genes and Genomes (KEGG) pathways in the monocytes of HC (cyan) and HLH (orange) donors, respectively. **(C)** Heatmap showing the regulatory potential of selected ligand molecules on the predicted target genes in the monocytes of HLH patients. Regulatory potentials were predicted according to the scRNA-seq data by NicheNet analysis. **(D)** Bar chart for comparing the abundance of each monocyte subtype between HC and HLH donors. Data are shown as mean±sem. *P*-values (t-test) for cell types with significant inter-groups difference are shown on top of the corresponding bars. **(E)** Network visualization of cell-cell communication through the SIGLEC1-SPN (SIGLEC1/CD169 and SPN/CD43) ligand-receptor pair between distinct cell subtypes in HLH donors. Distinct cell subtypes represented by circles with different colors are connected by arrows reflecting the interaction direction with color corresponding to the originating cell subtype. **(F)** Violin and box plots for comparing the anti-inflammatory (top) and pro-inflammatory (bottom) functional scores of monocyte subtypes between HC and HLH donor groups. For the box plots, the outlines of the boxes represent the first and third quartiles, the line inside each box represents the median, and boundaries of the whiskers are found within 1.5 times the interquartile range, with dots representing outliers. The same applies to all box plots in this paper, unless stated otherwise. **p < 0.01, ****p < 0.0001. Wilcoxon Rank Sum test (two-sided). **(G)** Dot plot for scaled expression of genes used for calculating the anti-inflammatory and pro-inflammatory functional scores in distinct monocyte subtypes of HC and HLH donor groups. **(H)** Line graphs for comparing the outgoing interaction strength (information flow) from distinct myeloid cell subtype through the selected signaling pathways between HC and HLH donor groups. **(I)** Dot plots showing TGF-β and IL-1β levels in culture supernatants from sorted CD14^+^ monocytes of HC and HLH donor groups, as measured by ELISA. (Independent-samples Student’s t-test).

Monocytes were markedly expanded in EBV-HLH PBMCs (*P* = 0.0004) (**Figure 1E**; **Supplementary Figure 1C**). Differential expression analysis revealed upregulation of genes involved in phagocytosis, proteasome activity and leukocyte migration, while genes related to pro-inflammatory signaling pathways, including NF-κB, Toll-like receptor, and TNF signaling were repressed (**Figures 3A, B**). Further, ligand to target gene interaction analysis nominated IFNA1, IFNA13, IFNG and IL10 as candidate upstream signals associated with SIGLEC1, CD86 and TGFB1 expression in monocytes. (**Figure 3C**). Detailed clustering demonstrated expansion of Mono_CD14_LGALS2, Mono_CD14_RTEN and Mono_CD14_NAMPT subtypes, with reduction of pro-inflammatory monocyte (Mono_CD14_IL1B) subtype (**Figure 3D**; **Supplementary Figure 3A**). Moreover, the monocyte subtypes in HLH patients were predicted to have ubiquitous interactions through the SIGLEC1-SPN ligand-receptor pair with the T, B and NK cells, which were not present in HCs (**Figure 3E**; **Supplementary Figure 3B**). Besides monocytes, neutrophils and cDCs exhibited similar transcriptional patterns (**Supplementary Figures 3C–E**). Functional scoring confirmed elevated anti-inflammatory and reduced pro-inflammatory signatures, with increased TGF-β pathway activity (**Figures 3F–H**; **Supplementary Figure 3F**). ELISA validation showed increased TGF-β secretion by CD14^+^ monocytes (**Figure 3I**).

### Exhaustion of T cells in EBV-HLH

**Figure 4:**
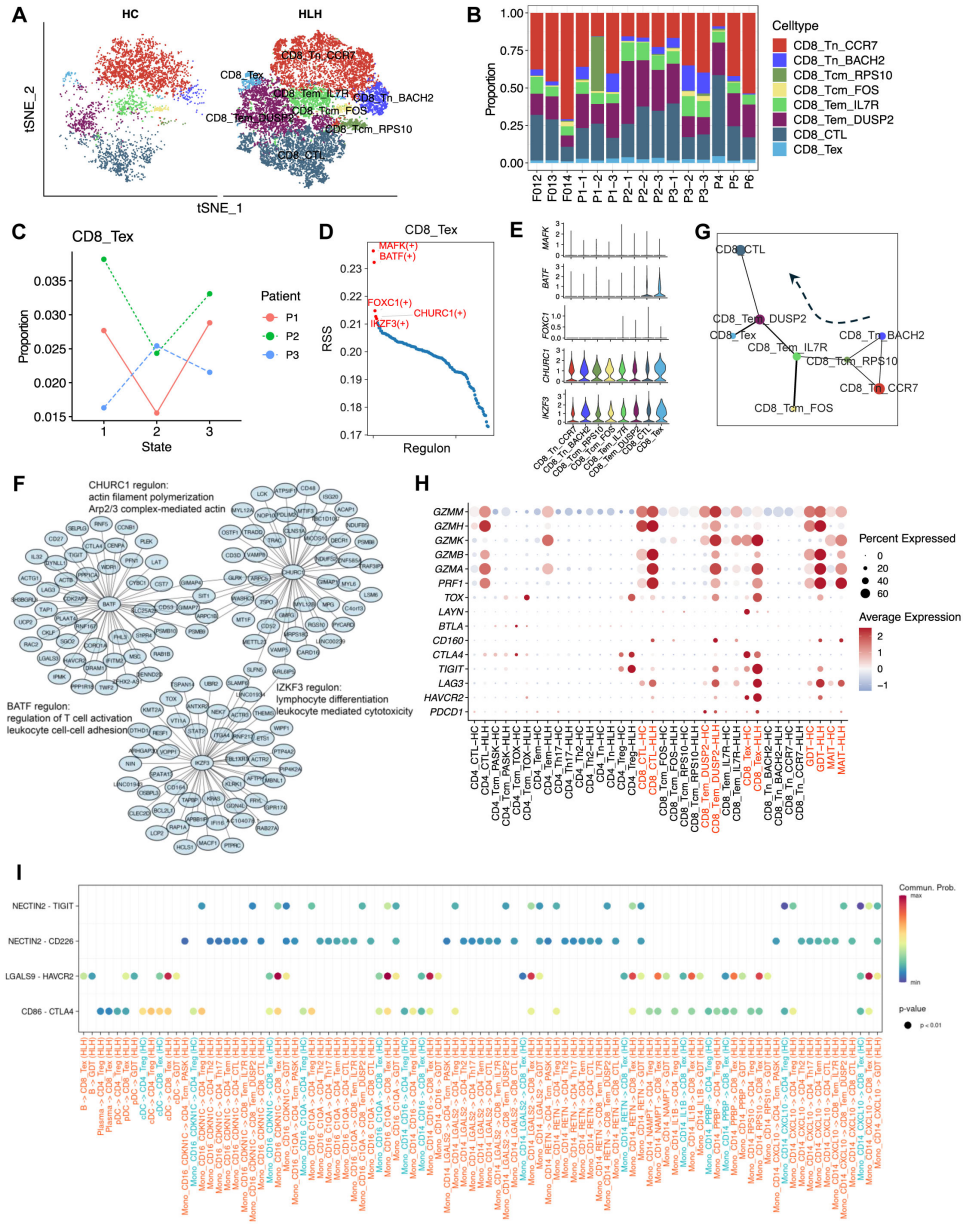
Characterization of T cells in PBMC of EBV-HLH patients. **(A)** tSNE plots of all CD8^+^ T cells, split by donor group (HC and HLH). Cells are color-coded according to subtype. **(B)** Stacked bar chart for summarizing the abundance of each CD8+ T cell subtype in each assayed sample. **(C)** Line graph showing the changes of abundance for CD8^+^ Tex cells in the assayed patients across different disease state (x-axis). **(D)** Scatterplot showing the predicted regulon specificity score (RSS, y-axis) and regulon activity ranking (x-axis) by distinct transcription factors (TFs). Top 5 TFs are shown in red, with ‘+’ in parentheses indicating upregulating effect. **(E)** Violin plots showing the expression distribution of selected TFs in distinct subtypes of CD8^+^ T cells of HLH patients. **(F)** Network visualization of the predicted BATF, CHURC1 and IZKF3 TF regulons. TFs and target genes are shown as ovals, with links in-between reflecting the regulatory relationship. Enriched Gene Ontology biological process (GO BP) terms for the target genes in each regulon are shown. **(G)** Networks for illustrating cell type transition relationship between different CD8^+^ T cell subtypes. Node size is proportional to cell population; edge width is proportional to transition potential. Dashed arrow depicts the transition direction from CD8^+^ naïve to cytotoxic T lymphocytes. **(H)** Dot plot for scaled expression of selected cell exhaustion and cytotoxicity related genes (x-axis) in distinct T cell subtypes (y-axis) of HC and HLH donor groups. Red font indicates the corresponding T cell subtypes are of higher exhaustion related gene expression in HLH patients than HCs. **(I)** Dot plot visualization of the selected co-inhibitory signal for T-cell subtypes of HLH and HC. The dot color and size represent the calculated communication probability and *p*-values, respectively.

Given the immunosuppressive property of myeloid cells on lymphocytes, we next investigate T cells with a particular focus on exhaustion. CD8^+^ T cells were subdivided into 8 subtypes (**Figures 4A, B**). HLH patients showed higher proportion of CD8_Tem_DUSP2, exhausted CD8^+^ T (CD8_Tex) and reduced population of naïve CD8^+^ T (CD8_Tn_CCR7) cells (**Figure 4B**; **Supplementary Figure 4A**). CD8_Tex were more abundant in patient P1 and P2 at the initial and relapse stages (**Figure 4C**). Consistent with our previous studies (26, 27), exhausted T cells in peripheral blood showed high expression of *ITGAE* and *CD38*, in addition to immune checkpoint-related genes (**Supplementary Figure 1E**). Similarly, CD4^+^ T cell analysis showed reduced naïve CD4^+^ T cells (CD4_Tn) and expansion of effector memory (CD4_Tem) and effector (CD4_CTL) subsets (**Supplementary Figures 4B–D**). FOXP3^+^ regulatory T cells were identifiable in the dataset; however, they did not show prominent expansion compared with other CD4^+^ T cell subsets (**Supplementary Figure 4**).

It was known that EBV-HLH is associated with CD8^+^ T cell exhaustion (28), we sought to explore the underlying mechanism. Exploratory SCENIC-based regulatory network inference nominated MAFK, BATF, FOXC1, CHURC1 and IKZF3 as candidate transcriptional regulators associated with the CD8_Tex state (**Figures 4D, E**). Further, the predicted target genes of BATF, CHURC1 and IZKF3 were functionally enriched for regulation of T cell activation, leukocyte cell-cell adhesion, actin filament polymerization, lymphocyte differentiation and leukocyte mediated cytotoxicity (**Figure 4F**). We then mapped the differentiation trajectory of CD8^+^ T cells in HLH, and the results suggested both cytotoxic CD8^+^ T (CD8_CTL) and CD8_Tex cells differentiated from the upstream CD8_Tem_DUSP2 cells which already expressed elevated immune checkpoint (e.g., *CTLA4*, *TIGIT*, *LAG3*, *HAVCR2* and *PDCD1*) (**Figures 4G, H**). Multiple T cell subsets displayed increased IFNG and TNFSF10 expression (**Supplementary Figure 4E**), with enhanced co-stimulatory and inhibitory receptor interactions, such as CD86-CD28, CD86-CTLA4, LGALS9-HAVCR2, and NECTIN2-TIGIT (**Figure 4I**; **Supplementary Figure 4F**). Moreover, NK/NKT-like cells in EBV-HLH showed stronger intercellular communication via the NECTIN2-CD226 axis which is associated with IFNG expression (29, 30).

Additionally, we identified specific subtypes of T cells potentially associated with disease progression. We defined more CD8_Tn_BACH2 in HLH (**Supplementary Figure 1E**) than HCs (**Supplementary Figure 4A**), especially at the relapse stage (**Supplementary Figures 5A, B**). In the CD4^+^ T cell, we identified less CD4_Tcm_PASK cells and more CD4_Tcm_TOX cells during relapse (**Supplementary Figures 1E**, **5C**). Further, expression of naïve T cell-associated genes (*CCR7* and *TCF7*) progressively decreased from CD4_Tn to CD4_Tcm_PASK and CD4_Tcm_TOX cells (**Supplementary Figure 1E**), suggesting that the CD4_Tcm_TOX cells differentiated from CD4_Tcm_PASK. CD4_Tcm_TOX cells also exhibited enriched senescence-related gene expression (**Supplementary Figures 5D, E**).

### TCR repertoire in EBV-HLH

**Figure 5:**
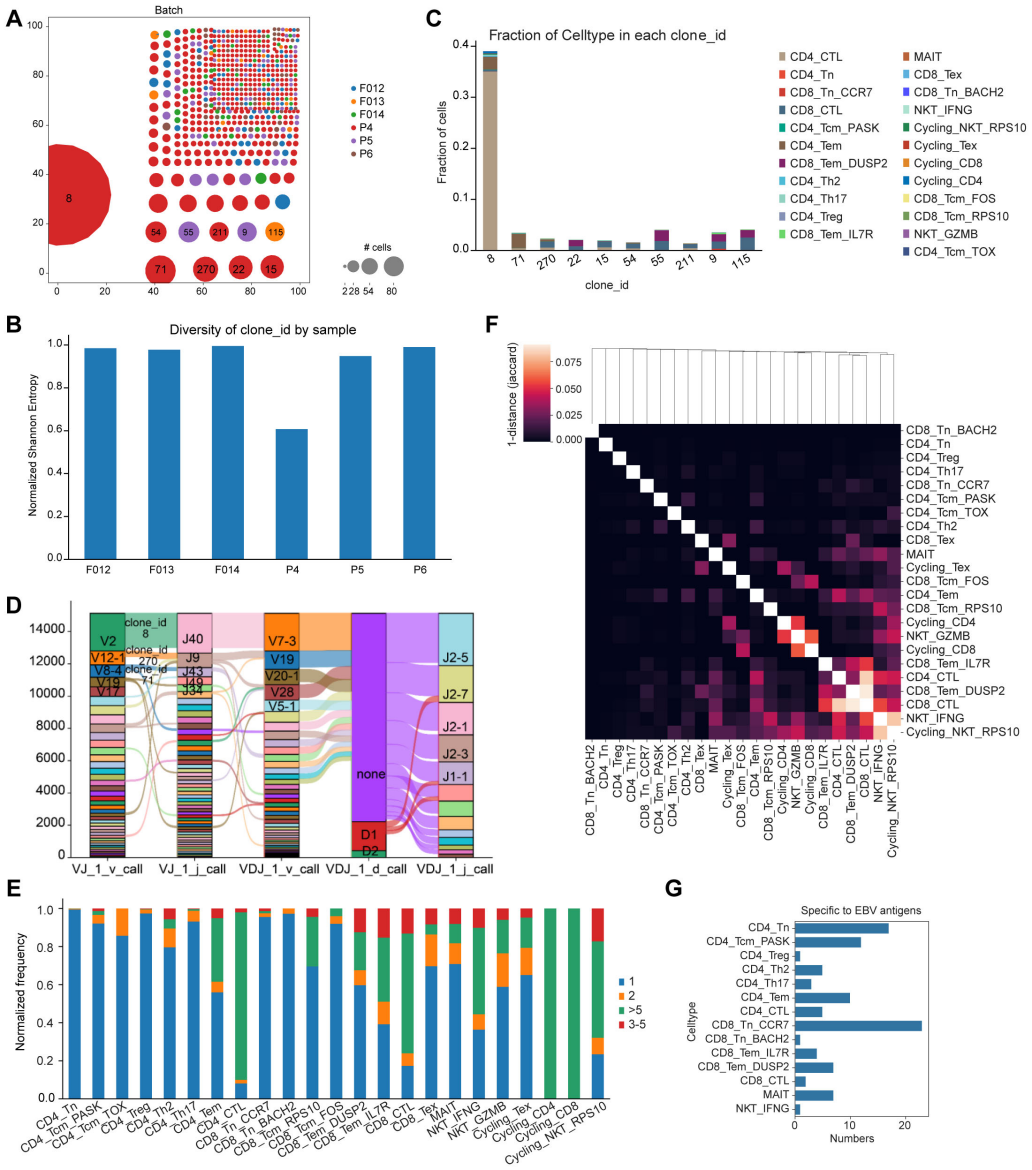
TCR repertoire characteristics in EBV-HLH patients. **(A)** Pie charts for TCR clones. Each pie represents a TCR clonotype, with size proportional to clone size. Color within each pie is proportional to the identification frequency of the TCR clonotype in the corresponding donor. Most of the pies are of single color, as the TCR clonotype is exclusive to one donor. The number on each pie is an arbitrary clonotype ID number. **(B)** Bar chart for comparing TCR repertoire richness (Normalized Shannon Entropy) between donors. Higher value indicates higher richness/diversity. **(C)** Stacked bar chart for summarizing the proportion of distinct subtype of T cells having the same TCR clonotype. Top 10 most frequent TCR clonotypes are shown. **(D)** Stacked bar chart for summarizing the usage frequency of distinct V, D and J genes in the identified TCRs. **(E)** Stacked bar chart for summarizing the relative TCR clone expansion frequency per T cell subtype (x-axis). **(F)** Heatmap showing the TCR repertoire similarity between distinct T cell subtypes. Higher value indicates higher similarity. **(G)** Bar chart for comparing the frequency of distinct subtype of T cells identified with TCR specific to EBV antigens.

scTCR-seq data was available for specific patients (P4, P5 and P6) and all three HCs (**Supplementary Figures 6A, B**). Expectedly, the HLH patients showed reduced TCR repertoire diversity especially for patient P4 (**Figures 5A, B**). Furthermore, individual TCR clonotype with high clonal expansion could be identified on diverse subtypes of T cells (**Figure 5C**). Although some V genes in TCRα and TCRβ could recombine with distinct J genes, specific V and J genes were more frequent than the others (**Figure 5D**).

Among the distinct subtypes of T cells, clonal expansion was mostly identified in effector cells and cycling T cells (**Figure 5E**; **Supplementary Figures 6C, D**). Further, the CD4_CTL, CD4_Tem, CD8_CTL, CD8_Tem_DUSP2 cells, and the NKT_IFNG and Cycling_NKT_RPS10 cells showed relatively high inter-cell cluster TCR clonotype similarity, reflecting the underlying differentiation relationship (**Figure 5F**). CD8_Tex cells shared similar TCR clonotypes with CD8_Tem_DUSP2 and Cycling_Tex cells (**Figure 5F**). Finally, T cells across various subtypes carried TCRs previously reported to be specific to EBV antigens (**Figure 5G**; **Supplementary Figure 6E**) but no particular subtype was enriched with EBV-specific TCRs.

## Discussion

HLH is a “cytokine storm” characterized by excessive systemic inflammation, but a specific immune profile capturing this complex dysregulation and the dynamic changes during treatment remains undefined. In this study, we combined longitudinal single-cell transcriptomic profiling with independent flow cytometric and ELISA validation to characterize the immune landscape of EBV-HLH and identified profound immune perturbations driven by EBV infection and activation. EBV transcript-positive NK/NKT-like cells exhibited clonal expansion and increased cytokine production, which may alter monocyte function and contribute to T-cell dysfunction, while FOXP3^+^ regulatory T cells were detectable but did not show prominent alterations. Collectively, these changes suggest a complex interplay between immune hyperactivation and immunosuppression, shedding light on potential mechanisms underlying EBV-HLH pathogenesis.

An irreversible decline in NK cell cytotoxicity is a major pathogenic mechanism of primary HLH (1, 4). However, EBV-HLH patients usually present with normal NK cell activity at diagnosis, which, even if initially reduced, often recovers after treatment and remains within the normal range during disease progression. Despite preserved NK cytotoxicity, the mechanisms underlying EBV persistence remain unclear. Carvelli et al. reported that NK cells from 68 sHLH patients exhibited activated phenotypes, normal cytotoxicity but lower cell number and IFN-γ production, which rendered NK cells ineffective in clearing EBV (31). In this study, we found that EBV transcript-positive NKT_IFNG, NKT_GZMB, and NK_FASLG cells were the main components of the pathological immune response, whereas EBV-uninfected NK_SELL and NK_CX3CR1 cells from the same EBV-HLH patients did not display these features. This highlights the pivotal role of EBV in driving specific immune responses in HLH pathogenesis. Interestingly, we identified one patient with EBV infection restricted to B cells who had a favorable outcome, suggesting that EBV infection of NK/NKT-like cells may initiate a dysregulated immune network associated with excessive IFN-γ production and elevated IL-10 responses. This observation is in line with findings from Liu et al., who compared EBV^+^, EBV^-^, and familial HLH cases and reported that EBV^-^ HLH was less frequently associated with T cell immunophenotypic abnormalities and cytokine dysregulation (28). Notably, in our cohort, EBV infection confined to B cells did not elicit excessive cytokine production and responded well to rituximab. Unlike Carvelli et al.’s findings, our data revealed significantly higher IFN-γ levels in EBV-HLH. IFN-γ was predominantly secreted by the EBV transcript-positive NK/NKT-like populations, and likely promoted further NKT-like cell activation via upregulation of the NECTIN2-CD226 axis (32, 33). Conversely, the simultaneously elevated NECTIN2-TIGIT signaling might suppress NK cell function, creating a complex regulatory environment. Although IFN-γ is recognized for its anti-tumor and antiviral properties, emerging evidence highlights its potential role in promoting immune evasion (34). This paradoxical immune scenario suggests a dual role for IFN-γ in EBV-HLH. A key factor in this regulation is CD160, which is essential for promoting IFN-γ production, while it also functions as an immune checkpoint molecule. In addition to PD-1 and LAG3 which were previously reported to be upregulated on exhausted CD8^+^ T cells in EBV-HLH (28), and CD160 may represent an additional inhibitory receptor associated with NK/NKT-like activation and T-cell dysfunction in EBV-HLH, although its functional role in this setting remains to be directly tested (25, 35). Elevated IL-10 levels and enrichment of IL-10-mediated JAK-STAT signaling in NK/NKT-like cells suggest that NK/NKT-like cells are responsive to the immunosuppressive IL-10 milieu (36, 37). This suggests a more nuanced dysfunction beyond a simple loss of NK activity, potentially involving a shift in NK subset dynamics.

Monocytes were significantly increased, as expected, in HLH patients (38, 39). These cells play diverse roles in inflammation, phagocytosis, and antigen presentation, yet their function in EBV-HLH appears skewed toward immune regulation rather than pathogen clearance. Tang et al. demonstrated that excessive IL-10 and IL-18 drive a hyperinflammatory state in mice by enhancing myelopoiesis and macrophage activation (38). While Suzuki et al (39*)*. compared NK/T-cell and B-cell EBV-HLH and highlighted dominant type I interferon responses. A recent study from Shen et al (40). also highlighted the role of monocytes in EBV-HLH pathogenesis. They identified a distinct population of IDO1^+^ monocytes associated with inflammatory activation and metabolic reprogramming through the kynurenine pathway in pediatric EBV-HLH. Consistent with these observations, our data also revealed prominent monocyte activation and extensive interactions between monocytes and lymphocyte populations. In our study, monocytes were activated by type II interferons, secreted predominantly by EBV transcript-positive NK and NKT-like cells, leading to an upregulation of CD169 (SIGLEC1) and CD86, which may enhance antigen presentation and activate tissue-resident macrophages (41, 42). Consistently, validation confirmed increased TGF-β production by monocytes from EBV-HLH patients. However, instead of a typical hyperinflammatory phenotype, the expanded monocyte subsets in EBV-HLH exhibited suppressed inflammatory pathways, indicating a shift toward an immunoregulatory state. Collins et al. (43) previously demonstrated an expansion of granulocytic myeloid-derived suppressor cells (G-MDSCs) in CAEBV and EBV-HLH, which potently suppressed T-cell proliferation driven by cytokines produced by EBV-infected T/NK cells. While the immunosuppressive myeloid population in our study consisted of CD14^+^ monocytes characterized by increased TGF-β and SIGLEC1 expression rather than phenotypically defined MDSCs. These findings further support a role for immunosuppressive myeloid cells in the pathogenesis of EBV-HLH. Additionally, we found that monocytes broadly interacted with T, B, and NK cells through the CD169 - CD43 interaction, which remains largely unexplored in EBV-HLH, may further contribute to immune dysfunction (44).

Immune exhaustion may contribute to the failure to clear EBV. Gao et al. found that immune cells in sHLH expressed higher levels of exhaustion markers, which impaired their ability to respond to EBV or control the inflammation (45). In our study, T cells displayed features of exhaustion, including upregulated immune checkpoint molecules and immunosuppressive cytokines. Consistent with Suzuki et al.’s observation of impaired CD8^+^ T cell responsiveness in EBV-HLH, exhaustion in our study primarily affected effector T cells, particularly CD8_Tem_DUSP2 and CD8_Tex subpopulation (39). Interestingly, exhausted T cells did not show evidence of dominant EBV-specific clonal expansion by TCR-seq. This observation raises the possibility that effector T cell dysfunction in EBV-HLH is driven primarily by cytokine-mediated inflammation rather than impaired antigen-specific TCR signaling. In this context, although FOXP3^+^ regulatory T cells were present, their limited expansion suggests that classical Treg-mediated suppression may not be the dominant mechanism underlying T cell dysfunction in EBV-HLH, consistent with the findings of Collins et al., who also found no increase in FOXP3^+^CD25^+^ Tregs in EBV-associated T/NK-cell lymphoproliferative disease. We further identified a distinct exhaustion profile marked by CD160 upregulation and concurrent IFN-γ and IL-10 signaling. In addition, CD4_CTL and CD4_Tem subpopulations increased despite the immunosuppressive environment in EBV-HLH, which raises the question of whether this expansion represents a compensatory mechanism or a response to elevated IFN-γ levels. Previous studies have suggested that IFN-γ could promote CD4^+^ CTL differentiation under inflammatory conditions (34). However, prolonged exposure to IFN-γ may exert dual effects, weaken the clearance of abnormal cells while drive effector T cell activation in a non-specific, cytokine-dependent manner rather than through direct EBV antigen recognition (34). Recent advances in EBV-HLH treatment have focused on targeted modulation of the hyperinflammatory response, including IFN-γ blockade with emapalumab and JAK-STAT pathway inhibition with ruxolitinib. Although these therapies have shown promising effects in controlling hyperinflammation, their efficacy is often limited (46–48). The potential biomarkers and mechanistic insights provided by this study may help refine future therapeutic strategies.

In summary, this study delineates a dysregulated immune landscape in EBV-HLH characterized by expanded and activated EBV transcript-positive NK/NKT-like cells, immunoregulatory monocytes, and exhausted T cells. Together, these findings support a hypothesis-generating model in which EBV-associated inflammatory states are accompanied by altered monocyte–lymphocyte communication, including NECTIN2-related signaling, and by activation/exhaustion programs in NK/NKT-like and T-cell populations. These predicted communication and regulatory pathways may contribute to excessive IFN-γ responses and progressive immune dysfunction, but further functional studies are required to determine whether they play causal roles in EBV-HLH pathogenesis. However, this study has certain limitations. First, the sample size is relatively small, and larger cohorts will be required to investigate subtype-specific immune characteristics. In addition, cross-dataset integration may leave residual technical effects or attenuate subtle biological variation; therefore, subtle group-level differences should be interpreted with caution. Second, although several candidate molecules and intercellular communication pathways were identified based on scRNA-seq data analysis, functional validation was limited to cytokine profiling and immune phenotyping, and further studies are required to validate the proposed signaling interactions in EBV-HLH pathogenesis.

## Data Availability

The datasets presented in this study can be found in online repositories. The names of the repository/repositories and accession number(s) can be found below: https://ngdc.cncb.ac.cn/gsa-human/browse/HRA010878, HRA010878.
