# Supplementary material for: Immune dysregulation and exhaustion in EBV- associated hemophagocytic lymphohistiocytosis: insights from single-cell transcriptomics and TCR repertoire analysis
Source: Front Immunol. 2026 Aug 5;17:1868027. doi: 10.3389/fimmu.2026.1868027 (PMC13485814; doi:10.3389/fimmu.2026.1868027)
Supplement: Supplementary file 1 [file Table1.docx]

| **Supplemental Table**  Supplemental Table 1. Demographic and Characteristics of Patients Based on HLH-2004 Diagnostic Criteria | | | | | | | | | | | | | | | |  |
| --- | --- | --- | --- | --- | --- | --- | --- | --- | --- | --- | --- | --- | --- | --- | --- | --- |
| Case | Sex/Age, y | Diagnosis | Fever  ≥ 38.5 °C | Splenomegaly | NEU 10^9^/L | HGB g/L | PLT 10^9^/L | Fib g/L | TG mmol/L | FER  µg/l | Hemopha-gocytosis | sCD25 U/ml | NK cell  activity % | Plasma  EBV-DNA Copies/ml | Genetic  defect |  |
| P1 | F/26 | CAEBV | + | + | 0.55 | 54 | 12 | 1.6 | 4.29 | 2174 | + | 6865 | 3.19 | 1.73E + 04 | Neg |  |
| P2 | M/25 | Vaccination-like T-cell lymphoma | + | + | 0.77 | 62 | 18 | 1.7 | 2.58 | 45159 | + | 162045 | NT | 1.53E + 04 | Neg |  |
| P3 | F/16 | CTD | + | + | 3.28 | 82 | 213 | 4.79 | 4.41 | > 8110 | + | NT | NT | 2.28E + 04 | Neg |  |
| P4 | M/22 | CAEBV | + | + | 7 | 65 | 714 | 1.27 | 3.01 | 226 | + | 4124 | 15.75 | 4.60E + 04 | Neg |  |
| P5 | M/39 | ANKL | + | + | 3.8 | 99 | 5 | 0.91 | 4.23 | 92150 | + | 66681 | 16.32 | 7.73E + 06 | TNFRSF13B |  |
| P6 | M/42 | CAEBV | + | + | 1.98 | 125 | 225 | 1.56 | 1.28 | 2283 | + | 3775 | 21 | 2.29E + 03 | Neg |  |
|  |  |  |  |  |  |  |  |  |  |  |  |  |  |  |  |  |
| *F* Female, *M* male, *CTD* Connective tissue disease, *CAEBV* Chronic active EBV infection, ANKL Aggressive NK-cell leukemia, *NEU* Neutrophi, *HGB* Hemaglobin, *PLT* Platelet, *Fib* Fibrinogen, *TG* Triglyceride, *FER* Ferritin, *sCD25* Soluable CD25, *ND* Not detected, *Neg* Negative | | | | | | | | | | | | | | | |  |
|  |  |  |  |  |  |  |  |  |  |  |  |  |  |  |  |  |

**Figure S1. PBMC scRNA-seq data quality assessment and identification of immune cell subtypes**

A. Violin plots of number of unique genes (left) and percentage of mitochondria genes (right) detected by scRNA-seq data in each sample.
B. PCA visualization of scRNA-seq data split by sample. The PCA plots showed no obvious batch effect in scRNA-seq data.

C-D. Stacked bar charts for summarizing the percentage (left) and absolute number (right) of cells identified as distinct major cell type.

E-G. Dot plots for scaled expression of selected marker genes in CD4^+^ T, CD8^+^ T, NK & NKT, cycling and monocyte cell subtypes.

**Figure S2. Additional characterization of NK and NKT cells in PBMC of EBV-HLH patients**

A. tSNE plot of all analyzed PBMCs per sample, color-coded according to whether EBV gene expression was detected in the corresponding cell. Red: EBV-positive; grey: EBV-negative.
B. Bar chart for summarizing percentage of EBV-positive cells per sample of patient P1, P2 and P3. Sample suffix (1, 2 or 3) corresponds to the disease state.
C. Heatmap showing the interaction probability of CD34-SELP signaling pathway among different cell subtypes in EBV-HLH patients. In the analyzed patients, CD34-SELP signaling is exclusively identified between HSC (sender) and Megakaryocyte (receiver) cells.
D. Violin and box plots for comparing the cell exhaustion (top) and cytotoxicity (bottom) functional score of NK and NK T cells between HC and HLH donor groups. *****p* < 0.0001. Wilcoxon Rank Sum test (two-sided).
E. Line graphs showing the changes of abundance for selected NK and NKT subtypes of PBMCs in patient P1 and P2 across different disease state (x-axis).
F. Dot plots showing the abundance for selected NK and NKT subtypes of PBMCs in patient P6 (left) and P5 (right).
G. Line graph showing the changes of abundance for B and plasma cells in the patient P3 across different disease state (x-axis).

**Figure S3. Additional characterization of myeloid cells in PBMC of EBV-HLH patients**

A. Stacked bar chart for summarizing the abundance of each monocyte subtype in each assayed sample.
B. Network visualization of cell-cell communication through the SIGLEC1-SPN ligand-receptor pair between distinct cell subtypes in HC donors. Distinct cell subtypes represented by circles with different colors are connected by arrows reflecting the interaction direction with color corresponding to the originating cell subtype.
C. Scatterplot for comparing average gene expression in the neutrophils of HC and HLH donors. Top 10 genes with significantly upregulated expression in HC and HLH are shown in cyan and orange, respectively.
D. Same as C, but for cDCs.
E. Bar chart for the significantly activated KEGG pathways in the cDCs of HC (cyan) and HLH (orange) donors, respectively.
F. Dot plot for scaled expression of selected cytokine genes (x-axis) in myeloid cell subtypes (y-axis). The last suffix in cell subtype name (HC or HLH) indicates the donor group from which the cells were analyzed.

**Figure S4. Additional characterization of T cells in PBMC of EBV-HLH patients.**

A. Bar chart for comparing the abundance of each CD8^+^ T cell subtype between HC and HLH donors. Data are shown as mean±sem. *P*-values (t-test) for cell types with significant inter-groups difference are shown on top of the corresponding bars.
B. tSNE plots of all CD4^+^ T cells, split by donor group (HC and HLH). Cells are color-coded according to subtype.
C. Stacked bar chart for summarizing the abundance of each CD4^+^ T cell subtype in each assayed sample.
D. Bar chart for comparing the abundance of each CD4^+^ T cell subtype between HC and HLH donors. Data are shown as mean±sem. *P*-values (t-test) for cell types with significant inter-groups difference are shown on top of the corresponding bars.
E. Dot plot for scaled expression of selected cytokine genes (x-axis) in T cell subtypes (y-axis). The last suffix in cell subtype name (HC or HLH) indicates the donor group from which the cells were analyzed.
F. Dot plot visualization of the selected co-inhibitory signal for NK and NKT cell subtypes of HLH and HC. The dot color and size represent the calculated communication probability and *p*-values, respectively.

**Figure S5. Characterization of specific T cell subtypes related to EBV-HLH**

A. Line graph showing the changes of abundance for CD8_Tn_BACH2 cells in the assayed patients across different disease state (x-axis).
B. Dot plot for KEGG pathway enrichments of specifically upregulated genes in CD8_Tn_BACH2 and CD8_Tn_CCR7 cells. Dot size is proportional to the fraction of genes annotated to the corresponding pathway over the total number of upregulated genes in the specific cell subtype.
C. Line graph showing the changes of abundance for CD4_Tcm_TOX cells in the assayed patients across different disease state (x-axis).
D. Same as B but for CD4_Tcm_TOX and CD4_Tcm_PASK cells.
E. Networks for illustrating cell type transition relationship between different CD4^+^ T cell subtypes. Node size is proportional to cell population; edge width is proportional to transition potential. Dashed arrow depicts the transition direction from CD4^+^ naïve to cytotoxic T lymphocytes.

**Figure S6. Additional characterization of TCR repertoire in EBV-HLH patients**

A. Stacked bar chart for the number of T cells identified with TCRs of complete αβ chains.
B. Stacked bar chart for the number of T cells identified with different form of TCR chains. VJ and VDJ corresponds to α and β chain, respectively.
C. Stacked bar chart for summarizing the absolute TCR clone expansion frequency per T cell subtype (x-axis).
D. Bar chart for comparing TCR repertoire richness (Normalized Shannon Entropy) between distinct T cell subtypes. Higher value indicates higher richness/diversity.
E. Bar chart for summarizing the number of T cells with TCRs specific to the listed antigens, according to VDJdb.
